# Hepatic and portal vein Doppler ultrasounds in assessing liver inflammation and fibrosis in chronic HBV infection with a normal ALT level

**DOI:** 10.3389/fmed.2023.1178944

**Published:** 2023-05-25

**Authors:** Li Tian, Shuyao Tang, Na Wang, Huan Deng, Qunxia Zhang, Tongdong Shi

**Affiliations:** ^1^Department of Infectious Disease, Institute for Viral Hepatitis, The Second Affiliated Hospital of Chongqing Medical University, Chongqing, China; ^2^Department of Ultrasound Medicine, The Second Affiliated Hospital of Chongqing Medical University, Chongqing, China

**Keywords:** chronic HBV infection, Doppler ultrasound, portal vein, hepatic vein, hemodynamics, blood flow spectra, liver fibrosis, ALT

## Abstract

**Aims:**

To discuss the clinical value of hepatic and portal vein Doppler ultrasounds in assessing liver inflammation and fibrosis in patients with chronic hepatitis B virus (HBV) infection, and a normal alanine transaminase (ALT) level.

**Methods:**

94 patients with chronic HBV infections who had undergone ultrasound-guided liver biopsies were enrolled and grouped by the liver tissue pathological results. Analyzed the differences and correlation between parameters of the hepatic and portal vein Doppler ultrasounds are discussed across different degrees of liver inflammation and fibrosis.

**Results:**

There were 27 patients with no significant liver damage and 67 patients with significant liver damage, there were significant differences in the parameters of the hepatic and portal vein Doppler ultrasounds between them (*p* < 0.05). As liver inflammation was aggravated, the inner diameter of the portal vein increased, and the blood flow velocities of the portal and superior mesenteric veins decreased (*p* < 0.05). When liver fibrosis became more severe, the inner diameter of the portal vein increased, while the blood flow velocities of the portal, superior mesenteric, and splenic veins decreased, and the Doppler waveforms of hepatic veins became unidirectional or flat (*p* < 0.05). The receiver operating characteristic (ROC) curve showed the assessment efficacy of hepatic and portal vein Doppler ultrasounds was superior to abdominal Doppler ultrasound alone in assessing liver fibrosis, and the combination of the two examination techniques outperformed any technique used alone.

**Conclusion:**

The hepatic and portal vein Doppler ultrasounds have important clinical value for assessing liver fibrosis in patients with chronic HBV infection, to aid improve the diagnosis of liver fibrosis.

## 1. Introduction

Hepatitis B virus (HBV) infection has become a heavy burden at the global level (1). There are approximately 257 million people with chronic HBV infection worldwide. Only 10.5% of HBV-infected people are diagnosed, and 17% of the diagnosed cases are properly treated (2). Currently, anti-HBV therapy remains the most effective approach against HBV infection (3). It has been demonstrated that this therapy can dramatically prolong the survival of patients with HBV-related liver cancer (4). HBV infection may evolve into hepatitis, cirrhosis, or even liver cancer. The progression of HBV infection through these stages usually goes undetected. The Guidelines for the Prevention and Treatment of Chronic Hepatitis B have expanded the indications for anti-HBV therapy (5). Nevertheless, there are a significant number of CHB patients who do not conform to the existing criteria and do not receive anti-viral therapy. Moreover, many such patients have disease progression or even die. It is therefore highly important to identify patients who do not have typical indications for anti-viral therapy but are still at risk for disease progression.

Non-invasive examination techniques are generally used to assess liver fibrosis and have received growing attention in recent years (6). Alanine transaminase (ALT) is a direct indicator of liver damage. It suggested the need to initiate anti-viral therapy when the ALT level exceeds the upper limit of normal (7). According to the histopathologic examination of the liver in some patients with chronic HBV infection and a normal ALT level, many of them already have varying degrees of inflammatory necrosis and fibrosis (8). Ultrasound is more acceptable to patients because there is no ionizing radiation exposure and the cost is low. Abdominal Doppler ultrasound has become a routine examination, but the diagnostic accuracy for early liver fibrosis remains low. Recent advances in color Doppler ultrasound can be used to measure the inner diameters and hemodynamics of the portal, superior mesenteric, splenic, and hepatic veins (9, 10). These Doppler parameters indirectly reflect the potential changes in the liver and compensate for the defects of the abdominal Doppler ultrasound, which only detects changes in liver morphology and parenchyma echogenicity. Unfortunately, this technique has rarely been used in clinical practice. We collected 94 patients with chronic HBV infection, a low viral load, a normal ALT level, and a normal FibroScan. These patients underwent hepatic and portal vein Doppler ultrasounds to analyze the correlations between the Doppler parameters of hepatic and portal veins and liver inflammation and fibrosis. The present study was intended to determine the effectiveness and applicability of hepatic and portal vein Doppler ultrasounds in predicting liver inflammation and fibrosis in patients with chronic HBV infection and a normal ALT level.

## 2. Materials and method

### 2.1. Patients

We preliminarily selected 230 patients with chronic HBV infections and persistently normal ALT levels, who had received regular follow-up evaluations at the Liver Disease Center at our hospital from 2018 to 2022 and satisfied the inclusion and exclusion criteria. Liver biopies were recommened for those patients with abnormal hepatic and portal vein color Doppler ultrasound tests, or those patients may clincally have potential pathological progress according to their desease history and other related examinations. Finally, a total of 94 patients had liver biopsies within 1 month, 67 of whom (approximately 71.3%) had significant liver damage (≥G2 and/or S2 based on pathologic examination).

### 2.2. Inclusion and exclusion criteria

1. The inclusion criteria were as follows: normal liver biochemical tests; HBV-DNA level ≤ 10^4^ IU/mL; FirbroScan <7.1 Kpa; normal ECG; and normal abdominal Doppler.
2. The exclusion criteria were as follows: combined with severe cardiovascular and respiratory diseases; renal function impairment; hematologic diseases; autoimmune diseases; pregnancy; or lactation. All subjects signed the informed consent form.

### 2.3. Methods

#### 2.3.1. Hepatic and portal vein Doppler ultrasound

The patients underwent hepatic and portal vein Doppler ultrasounds 12–24 weeks before the liver biopsies using a Sonoscape S60 ultrasound machine (Shenzhen, China). All patients underwent ultrasound examinations by a fixed group of 2–3 ultrasound specialists who were highly responsible and had extensive experiences in the field of abdominal ultrasonography, and blinded to the clinical and biochemical profiles of patients. All patients scanned following an overnight fast of at least 6-8 h to reduce excess bowel gas that may obscure the vascular structures.

**Figure 1:**
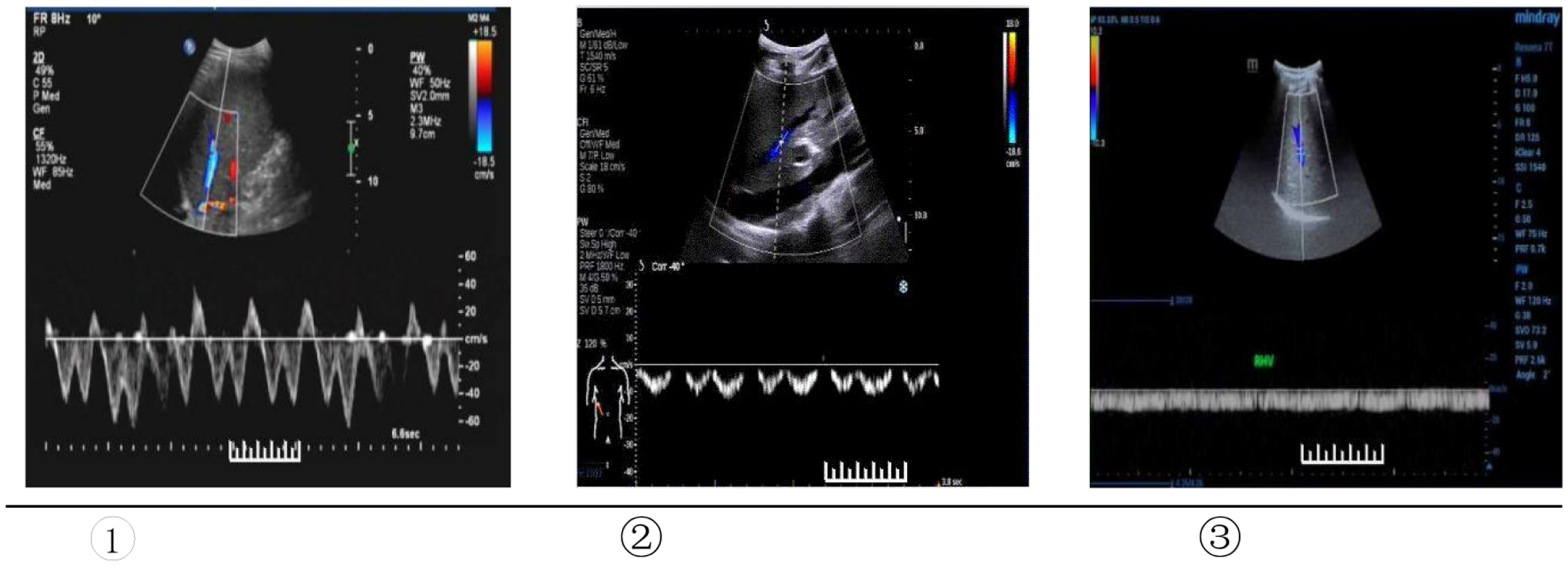
Type of Doppler waveforms of the hepatic veins. ① Triphasic waveform. ② Biphasic waveform. ③ Flat waveform.

1. The Doppler ultrasound scan of the portal venous system: with the patient in the supine position, B-mode ultrasound of the hepatic parenchyma was done first to assess the liver span, echogenicity, echotexture, and surface nodularity. The inner diameters and blood flow velocities were then measured in the portal, superior mesenteric, and splenic veins. The portal vein was measured at the main trunk of the portal vein where is the confluence of the portal vein and a point 1.0–2.0 cm away from the first porta of liver, and the superior mesenteric vein was measured at 1.0 cm in front of the confluence of the superior mesenteric vein, the splenic vein was measured at a distance of 1.0–2.0 cm from the splenic hilum. The intersection angle between the long axis of the blood vessel and the color Doppler ultrasonography was controlled to below 60°.
2. Hepatic vein Doppler ultrasound scan: the patient was instructed to keep the breathing calmly. The 3.5 MHz convex probe was placed under the right costal margin, and the oblique section was scanned upward. When the middle hepatic vein is clearly displayed, Pulsed Doppler evaluation of HV was then performed at 5 cm from the second hepatic portal with PW mode, and record the pulse Doppler flow curve, namely the blood flow spectrum. We adopted the classification of the hepatic vein Doppler waveforms proposed by Koizumi et al. (11), as follows: (1) Bidirectional triphasic, or bidirectional tetra phasic waveforms (the first forward wave is the A-wave, the second backward wave is the S-wave, and the second backward wave is the D-wave); (2) Biphasic waveform; the decreased amplitude of the phasic oscillations without the short phase of reversed flow; (3) Monophasic or flat waveform. Type ① was considered normal, and the type ② and ③ were abnormal (Figure 1).

#### 2.3.2. Histopathologic examination by liver biopsy

All patients completed preoperative routine blood tests, blood biochemistry tests, coagulation function tests, and abdominal Doppler ultrasound to exclude contraindications for a liver biopsy. All of the patients signed an informed consent form. To minimize the deviation as much as possible, in this study, liver biopsy was performed by one fixed physician who had long been engaged in the diagnosis and treatment of liver diseases and was assisted by a fixed ultrasound technician. They all received professional training and have many years of professional experiences. Bedside ultrasound-guided liver biopsies (using an 18 G biopsy needle) were performed to collect liver samples measuring 1.5–2.2 cm in length. The samples were fixed in 4% formaldehyde, embedded in paraffin, sectioned into 5-um slices, and stained with HE and Masson’s. The histopathological changes were scored by a fixed group of liver pathologists and confirmed by one superior chief liver pathologist. According to international consensus (12), liver inflammation and fibrosis were classified into stages 0–4. The more advanced the stage, the more severe the liver inflammation and fibrosis. Significant liver damage was defined as liver inflammation (≥G2) of moderate severity and above and/or liver fibrosis (≥S2) of moderate severity and above based on pathologic examination.

### 2.4. Observation indicators

The observation indicators were as follows:

1. The clinical data of liver biopsy;
2. The hepatic and portal vein Doppler ultrasounds and liver biopsy;
3. Correlation and binary logistic regression analysis between the hepatic and portal vein Doppler ultrasound parameters and liver biopsy;
4. The diagnostic efficacy, cut-off values, and sensitivity and specificity of abdominal Doppler ultrasound plus hepatic and portal vein Doppler ultrasounds in assessing liver fibrosis.

### 2.5. Statistical methods

The data were processed and analyzed using SPSS 25.0 software. Measurement data obeying (or approximately obeying) a normal distribution are expressed as the mean ± standard deviation (X ± S). Enumeration data are expressed as *n*% and analyzed using a *t*-test and chi-square test. Measurement data not obeying a normal distribution are expressed as medians and interquartile range [M (p25–p75)]. Two group comparison of such data was made using the Mann–Whitney *U*-test. The ordered categorical variables were made using the Jonckheere-Terpstra test and the Cochran-Armitage test. The diagnostic efficacy of the hepatic and portal vein Doppler ultrasounds for liver fibrosis was assessed using the Spearman correlation test, binary logistic regression analysis, and receiver operating characteristic (ROC) curve. A *p* < 0.05 indicated a significant difference.

## 3. Result

### 3.1. The clinical data of liver biopsy.

Grouping based on liver histopathology: there were 27 patients with no significant liver damage and 67 patients with significant liver damage. The hepatic and portal vein Doppler ultrasounds showed that the inner diameter of the portal and splenic vein in the Group significant liver damage were wider than in Group no significant liver damage, the blood flow velocity of the portal and superior mesenteric vein were more slowly than in Group no significant liver damage; The waveforms of abnormal hepatic vein (biphasic or flat waveform) were more than in Group no significant liver damage, and the differences were statistically significant (*p* < 0.05; Table 1). The two groups did not differ significantly concerning gender, age, HBeAg, family medical history, ALT, AST, HBV-DNA level, and FibroScan (*p* > 0.05; Table 1).

**Table 1:** Comparison of clinical data between the groups with and without significant liver damage.

Parameters	No significant liver damage (27) (<G2/S2)	Significant liver damage (67) (≥G2/S2)	Test value	*p*-value
Age (years)	34 (30,44)	37 (30,46)	0.753	0.455
Gender (*n*%)				
Male	20 (74.07%)	48 (71.64%)	0.57	0.811
Female	7 (25.93%)	19 (28.36%)		
HBeAg (*n*%)				
(+)	9 (33.3%)	22 (32.8%)	0.002	1.000
(−)	18 (66.7%)	45 (67.2%)		
Family medical history (*n*%)				
Yes	10 (37%)	23 (34.3%)	0.062	0.815
No	17 (63%)	44 (65.7%)		
ALT (U/L)	26.96 ± 8	28.22 ± 8	−5.25	0.603
AST (U/L)	25 (23,29)	28 (22,30)	−3.125	0.444
HBV-DNA log (IU/mL)	3 (2,4)	3 (2.3,4.19)	−0.363	0.175
Fibroscan (kPa)	4.8 (4.5,5.8)	5.1 (4.7,5.8)	−1.100	0.274
Inner diameter of the portal vein (mm)	10 (10,12)	11 (10,13)	2.801	0.005*
Blood flow velocity of the portal vein (cm/s)	25.1 (20.3,29.2)	17.4 (15.7,20)	−3.748	<0.001*
Inner diameter of the superior mesenteric vein (mm)	7 (6,8)	8 (7,9)	1.758	0.079*
Blood flow velocity of the superior mesenteric vein (cm/s)	22 (17.1,30)	18.1 (15.5,23)	−2.512	0.011*
Inner diameter of the splenic vein (mm)	5 (5,7)	6 (5,7)	2.029	0.042
Blood flow velocity of the splenic vein (cm/s)	18 (14.4,21)	17.3 (15.5,22)	−0.572	0.571
Doppler waveform of the hepatic veins (*n*%):				
Normal	16 (59.26%)	16 (23.88%)	10.728	0.002*
Abnormal	11 (40.74%)	51 (76.12%)		

^*^p < 0.05 indicates a significant difference.

### 3.2. The hepatic and portal vein Doppler ultrasounds and liver biopsy

#### 3.2.1. Doppler ultrasounds and liver inflammation

**Figure 2:**
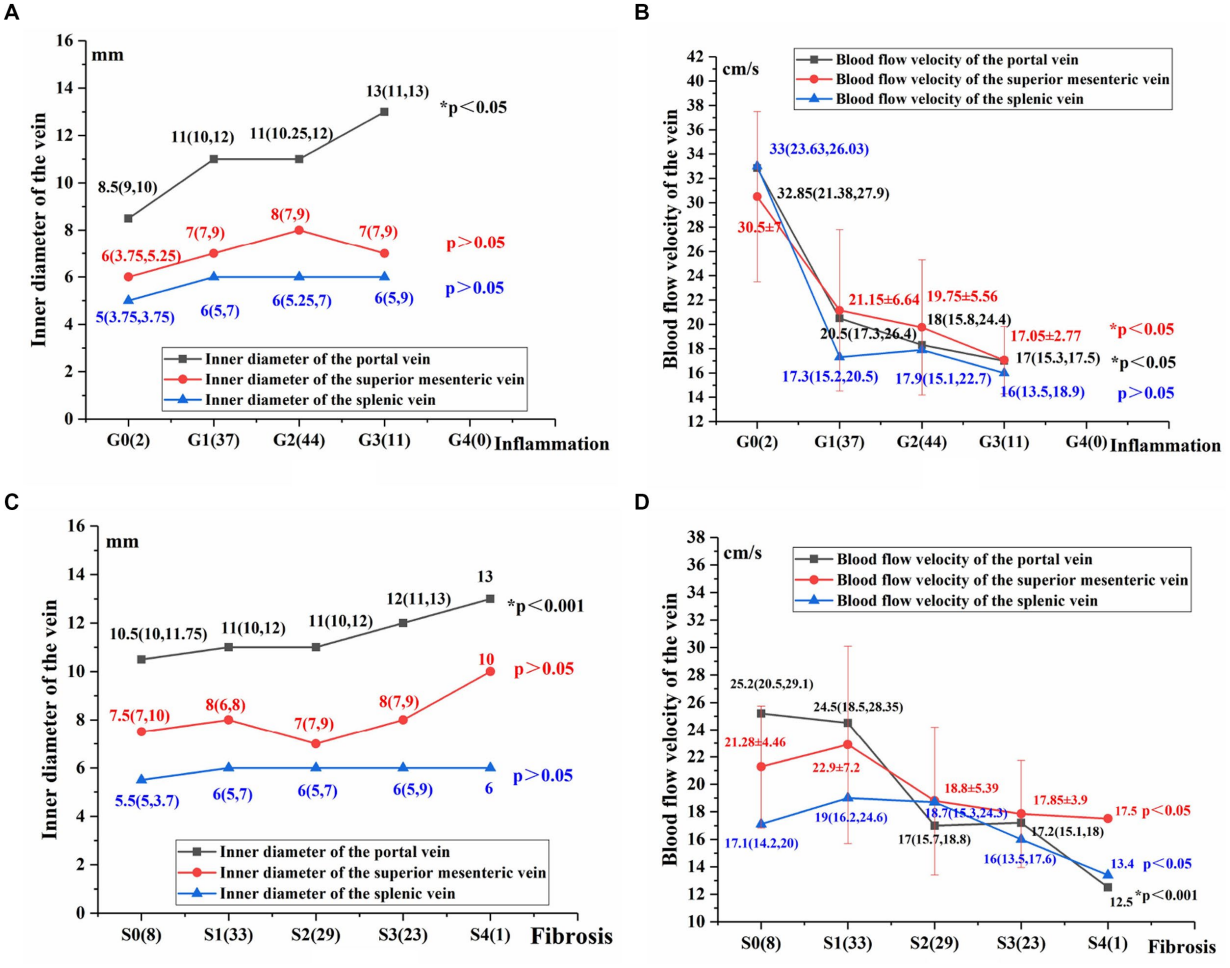
Comparison of hepatic and portal vein Doppler ultrasound parameters between varying degrees of liver inflammation and fibrosis **(A–D)**. Using the Jonckheere-Terpstra test, *p < 0.05 indicates a significant difference.

The inner diameter of the portal vein (mm): G3>G2>G1>G0, increased with the aggravation of liver inflammation (*p* < 0.05; Figure 2A). The blood flow velocity of the portal vein and superior mesenteric vein (cm/s): G3<G2<G1<G0，decreased as liver inflammation became more severev (*p* < 0.05; Figure 2B).

The following parameters did not differ significantly across patients with varying degrees of liver inflammation: the inner diameter of the superior mesenteric vein (mm), the inner diameter of the splenic vein (mm), the blood flow velocity of the splenic vein, and type of the hepatic vein Doppler waveforms (*P* > 0.05; Figures 2A,B).

#### 3.2.2. Doppler ultrasounds and liver fibrosis

The innner diameter of the portal vein (mm): S4 > S3 > S2 > S1 > S0, increased as liver fibrosis became more severe (*p* < 0.05; Figure 2C). The inner diameter of the superior mesenteric vein and the splenic vein (mm) did not differ significantly in patients with varying degrees of liver fibrosis (*p* > 0.05; Figure 2C).

The blood flow velocity of the portal vein (cm/s): S4 < S3 < S2 < S1 < S0. The blood flow velocities of the superior mesenteric vein and splenic vein (cm/s): S4 < S3 < S2 < S1/S0. The blood flow velocities of the portal, superior mesenteric and splenic veins decreased as liver fibrosis became more severe (*p* < 0.05; Figure 2D).

**Figure 3:**
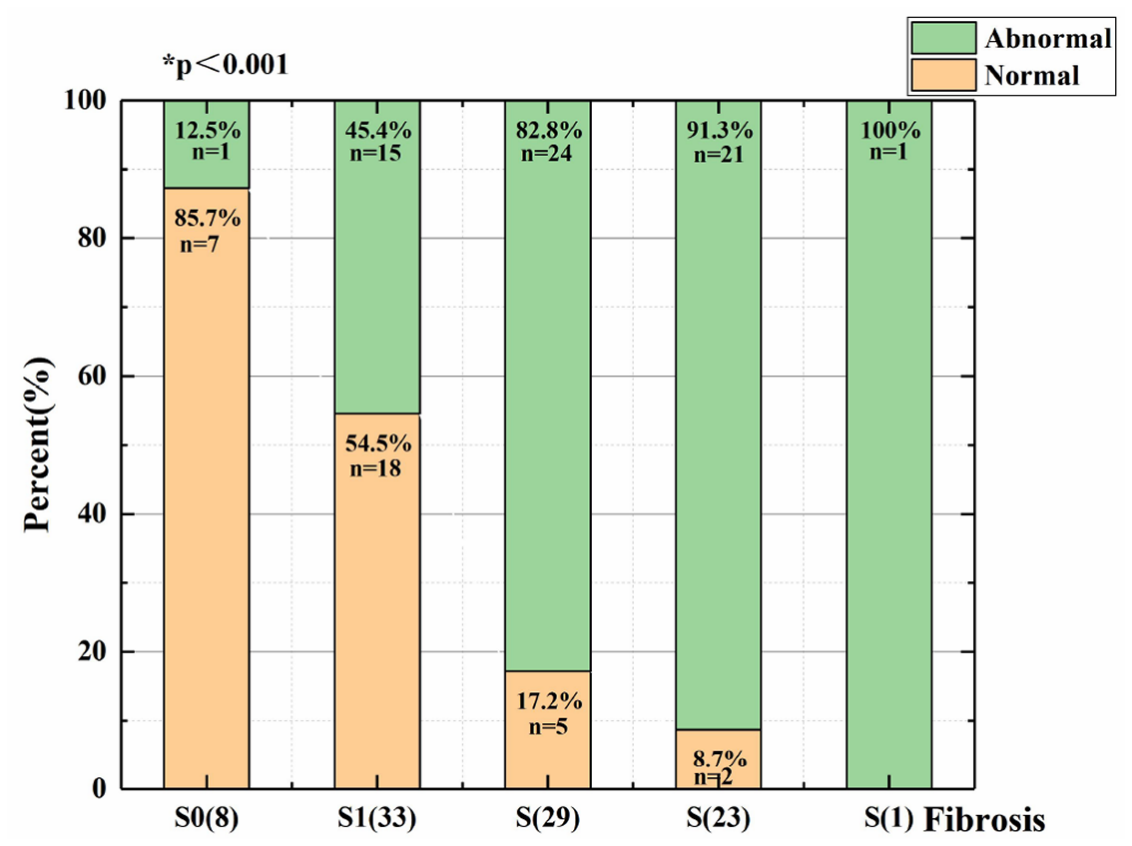
Distribution of patients with varying degrees of liver fibrosis and different types of hepatic vein Doppler waveforms.

As liver fibrosis became more severe, the percent of abnormal waveforms increased, the waveforms of the hepatic veins became unidirectional or flat, and the Cochran-Armitage test shows that the difference was statistically significant in patients with varying degrees of liver fibrosis (*p* < 0.05; Figure 3).

### 3.3. Correlation and binary logistic regression analysis between the hepatic and portal vein Doppler ultrasound parameters and liver biopsy

Spearman correlation test was performed to assess hepatic and portal vein Doppler ultrasound parameters and the degree of liver inflammation and fibrosis. The inner diameter of the portal vein and waveforms of hepatic vein was positively correlated with the degree of liver inflammation and fibrosis. The blood flow velocity of the portal, superior mesenteric, was negatively correlated with the degree of liver fibrosis (*p* < 0.05, Table 2).

**Table 2:** Correlation between the hepatic and portal vein Doppler ultrasound and the liver tissue pathology.

Doppler ultrasound parameters	Inflammation		Fibrosis	
	roh	*p*	roh	*p*
Inner diameter of the portal vein (mm)	0.318	0.002*	0.359	<0.001*
Blood flow velocity of the portal vein (cm/s)	−0.325	0.001*	−0.534	<0.001*
Blood flow velocity of the superior mesenteric vein (cm/s)	−0.253	0.015*	−0.299	0.003*
Waveforms of hepatic vein	0.215	0.037*	0.515	<0.001*

*p < 0.05 indicated a significant difference.

Relevant indicators were included for binary logistic regression analysis. Hemodynamic changes of the liver, as characterized by the blood flow velocity of the portal veins and hepatic vein waveform, could be used as predictors of the risk factors for significant liver fibrosis in chronic HBV infection with a normal ALT level (*p* < 0.05, Table 3).

**Table 3:** Binary logistic regression analysis to identify the risk factors for liver fibrosis in patients with chronic HBV infection and a normal ALT level.

	Correlation coefficient	Wald-value	*p*-value	*OR*	95% confidence interval	
Blood flow velocity of the portal vein (cm/s)	−0.180	7.719	0.005*	0.835	0.736	0.948
Waveforms of hepatic vein (Biphasic waveform)	1.77	7.202	0.007*	5.912	1.615	21.644

*p < 0.05 indicated a significant difference.

### 3.4. The diagnostic efficacy, cut-off values, and sensitivity and specificity of abdominal Doppler ultrasound plus hepatic and portal vein Doppler ultrasounds in assessing liver fibrosis

**Figure 4:**
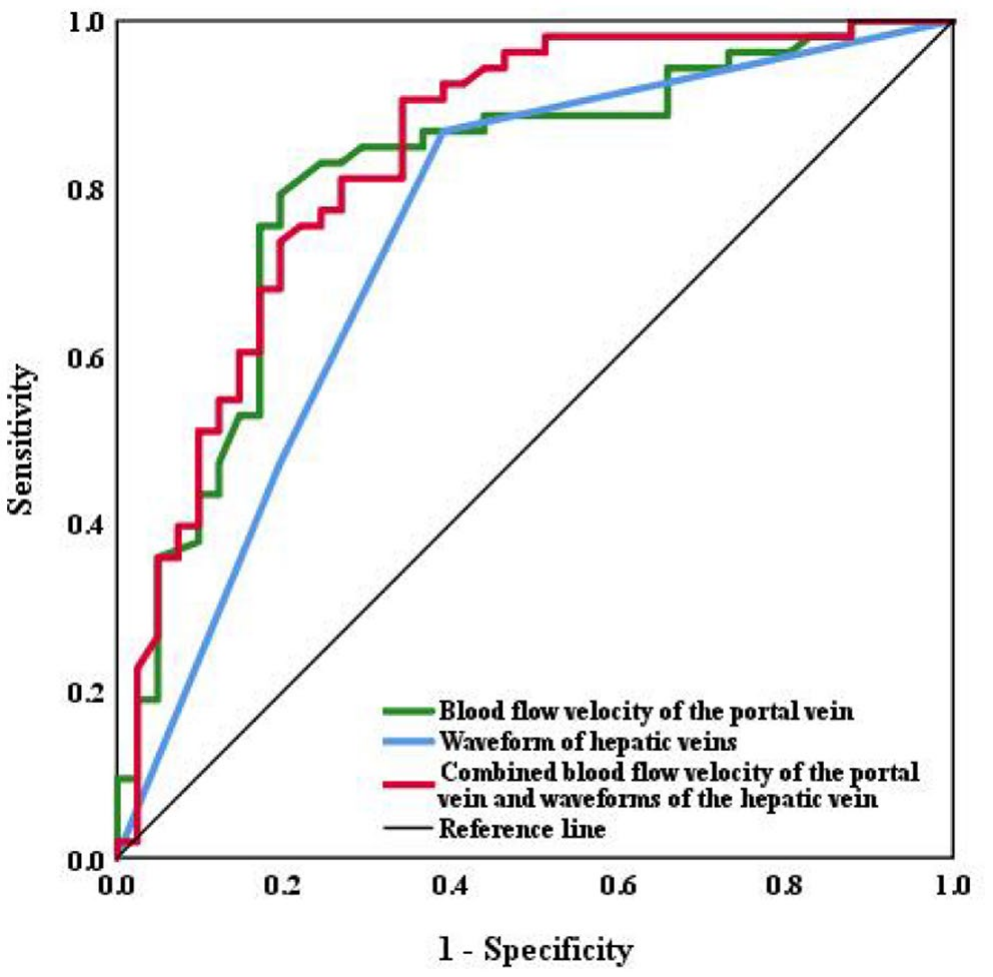
ROC curve of the hepatic and portal vein Doppler ultrasounds and abdominal Doppler in assessing liver fibrosis.

The area under the ROC curve was analyzed with the following results: combined blood flow velocity of the portal vein and waveforms of the hepatic vein (AUC = 0.837, *p* < 0.001) > Blood flow velocity of the portal vein (AUC = 0.812, *p* < 0.001) > Waveforms of the hepatic veins (AUC = 0.746, *p* < 0.001). It shows that significant liver fibrosis may existed in liver when the blood velocity of the portal vein is <18.75 cm/s, or the hepatic vein waveform becomes unidirectional or flat waveform. The results also indicate that the sensitivities (86.8%) of the waveform of hepatic veins is higher than the blood flow velocity of the portal vein (79.2%), while the blood flow velocity of the portal vein joint with hepatic waveforms proves the highest sensitivity (90.6%). The respective cut-off values, sensitivities, and specificities are shown in Table 4 and Figure 4.

**Table 4:** Assessment efficacy of hepatic and portal vein Doppler ultrasounds and abdominal Doppler in grade S2 and above liver fibrosis.

Assessment efficacy in liver fibrosis	AUC (95% confidence interval)	*p*-value	Cut-off	Sensitivity (%)	Specificity (%)
Blood flow velocity of the portal vein (cm/s)	0.812 (0.722–0.903)	<0.001*	18.75(cm/s)	79.2	80.5
Waveform of hepatic veins	0.746 (0.642–0.851)	<0.001*	Biphasic or flat waveform	86.8	61
Combined blood flow velocity of the portal vein and waveforms of the hepatic vein	0.837 (0.752–0.922)	<0.001*	The velocity ≤ 23.58(cm/s), and biphasic or flat waveform	90.6	65.9

*p < 0.05 indicated a significant difference.

## 4. Conclusion

Liver fibrosis usually results from prolonged liver inflammation, in which normal liver tissues are replaced by fibrotic tissues and regenerative nodules. Liver fibrosis may eventually progress into cirrhosis (13). Early liver fibrosis and cirrhosis can be reversed by appropriate treatments. Fibrous scars are usually generated by myofibroblast activation associated with chronic liver damage, which leads to extracellular matrix protein secretion, such a process can result in liver fibrosis (14–16). These changes will give rise to the non-uniform thickness of intra-hepatic and extra-hepatic vessels and abnormal blood flow velocity and orientation. Then the intrahepatic vascular resistance increases and extensive portal collateral circulation is formed, which further causes portal hypertension (17). It remains challenging to detect liver fibrosis (cirrhosis) at an early stage due to the hidden onset of cirrhosis. No uniform standard has been developed, despite a great variety of non-invasive diagnostic techniques that have emerged in recent years (18). Liver specialists have long been troubled by the lack of convenient, the non-invasive diagnostic technique for patients with chronic HBV infection who receive outpatient clinic follow-up for an extended period, have a normal ALT level, a normal FibroScan, normal abdominal Doppler ultrasound, and a low viral load. Indeed, early detection of liver fibrosis is highly important for early initiation of anti-HBV treatment, if indicated.

In the present study, hepatic and portal vein Doppler ultrasounds were performed to measure the inner diameters of hepatic and portal veins and the changes in blood flow velocity of the hepatic veins as indicators of hemodynamics change. The purpose of the current study was to non-invasively assess disease activity in patients with liver inflammation and/or the degree of liver fibrosis. Among 230 patients with chronic HBV infection, a normal ALT level, and a low viral load, 94 had liver biopsies. Significant liver damage was detected in 71.3% of the patients. It was initially found that the hemodynamic parameters of the significant liver damage group were significantly different from those in the group with no significant liver damage. We further analyzed the changes in the parameters of hepatic and portal vein Doppler ultrasounds between varying degrees of liver inflammation and fibrosis: as the liver inflammation and fibrosis were aggravated, the inner diameters of the hepatic veins increased. The blood flow velocities of the portal, superior mesenteric, and splenic veins decreased as liver fibrosis worsened. These changes were mainly related to liver fibrosis and consistent with the findings by Hui et al. (19). The possible reasons include hepatic cell necrosis, massive fibrous tissue hyperplasia, perisinusoidal fibrosis or hepatic sinusoidal obstruction, pseudolobular formation, and compression of intrahepatic venules. As the blood outflow from the hepatic sinuses is obstructed, intrahepatic vessels will become distorted and blocked. The portal vein circulation is limited, resulting in blood stasis, decreased blood flow velocity, and a widened portal vein. The superior mesenteric vein, splenic vein, and other collateral vessels will finally be involved.

Sudhamsku et al. (20) reported that hepatic hemodynamic changes and abnormal intrahepatic vascular shunts cause changes in the Doppler waveforms of the hepatic veins. The hepatic veins drain the liver into the inferior vena cava, then the blood flow enters the right atrium. Because the hepatic vein blood flow is influenced by cardiac systole and diastole, the Doppler waveforms of the hepatic veins are consistent with the heart, being either triphasic or tetra phasic. Liver fibrosis causes the narrowing of the hepatic veins and a decrease in elasticity and compliance, and the Doppler waveforms of the hepatic veins will change accordingly (19). Soroida et al. (21) quantified the Doppler waveforms of the hepatic veins instrumentally, based on the predicted degree of liver fibrosis in patients with chronic liver disease. We observed that the Doppler waveforms of the hepatic veins became unidirectional or flat in 76.1% of the patients with significant liver fibrosis using a color Doppler ultrasound device. This fact confirmed the clinical value of Doppler waveforms of the hepatic veins for assessing the degree of liver fibrosis. Finally, the blood flow velocities in the portal and superior mesenteric veins, and hepatic vein waveforms were included in the binary logistic regression analysis. We compared three methods of ultrasonography (including Abdominal Doppler Ultrasound, and hepatic and portal vein Doppler ultrasounds) and plotted ROC curves. When the blood flow velocity of the portal vein is less than 18.75 cm/s, it is more possible to occur significant liver fibrosis; And the hepatic and portal vein Doppler ultrasounds outperformed abdominal Doppler ultrasound alone in the assessment efficacy for liver fibrosis. In addition, the combined hepatic and portal vein Doppler ultrasounds were superior to either alone.

Taken together, patients with chronic HBV infection, a normal ALT level, and a low viral load might also be complicated by significant liver damage. In the present study, hepatic and portal vein Doppler ultrasounds were performed to measure the inner diameters of hepatic and portal veins and the changes in blood flow velocity in the hepatic veins to reflect the hemodynamics change. Compared with abdominal Doppler, this auxiliary technique could conveniently and non-invasively detect liver inflammation and fibrosis at an early stage.

## Data availability statement

The raw data supporting the conclusions of this article will be made available by the authors, without undue reservation.

## Ethics statement

The studies involving human participants were reviewed and approved by the Ethics Committee of the Second Affiliated Hospital of Chongqing Medical University. All subjects signed the informed consent form.

## Author contributions

LT and TS made the concept and design of the study. LT designed and wrote the manuscript. LT and ST analyzed the data and screened the literature and collected data. NW, HD, QZ, and TS critically revised the manuscript. All authors have read and agreed to the published version of the manuscript.

## Funding

This work was supported by the National Natural Science Foundation of China (No: 82002597).

## Conflict of interest

The authors declare that the research was conducted in the absence of any commercial or financial relationships that could be construed as a potential conflict of interest.

## Publisher’s note

All claims expressed in this article are solely those of the authors and do not necessarily represent those of their affiliated organizations, or those of the publisher, the editors and the reviewers. Any product that may be evaluated in this article, or claim that may be made by its manufacturer, is not guaranteed or endorsed by the publisher.
